# The mental health of health workers in the pandemic 

**DOI:** 10.2471/BLT.21.020621

**Published:** 2021-06-01

## Abstract

Increased readiness to discuss sensitive topics will play a key role in alleviating the psychological stress on health workers responding to the coronavirus disease 2019 (COVID-19) pandemic. Andréia Azevedo Soares reports.

For Dr Flávia Machado, what happened in Manaus was a wake-up call. Head of the intensive care unit at the Federal University of São Paulo, Brazil, Machado watched the situation unfold in the capital of Amazonas state, north-western Brazil, with a mixture of alarm and disbelief. 

“The spike in COVID-19 cases back in January exposed a severe lack of medical oxygen in the system,” she says. “It really brought home to me and my team the possibility that we too might be receiving patients we cannot treat.” 

Machado says that if nothing changes her team could soon be without the sedatives and neuromuscular blocker agents it needs to ventilate coronavirus 2019 (COVID-19) patients. “This is the worst situation we have ever faced,” she adds. “And it is causing a lot of anxiety, depression and sleepless nights.” 

What is true of Machado and her team is true for health professionals worldwide. While accounts of health systems pushed beyond their limits by the pandemic have tended to focus on the plight of patients and their families, health workers are suffering too.

“Health workers are being subjected to strains that include but are by no means limited to working long hours, sometimes without appropriate personal protective equipment, increased exposure to patient suffering and death,” says Dr Aiysha Malik, a mental health expert working in the Department of Mental Health and Substance Use at the World Health Organization (WHO).

The impact such stresses are having on health professionals is beginning to emerge in research that includes a cross-sectional study published in *JAMA Network Open* which revealed that 71.5% (899 of 1257 participants) of health workers treating COVID-19 patients in Chinese hospitals reported psychological distress, while around half reported symptoms of depression and anxiety. Just over a third (427 participants) reported suffering symptoms of insomnia. 

A study published in the May issue of the *European Journal of Psychotraumatology* paints a similar picture. Performed right after the first wave of COVID-19 in the United Kingdom of Great Britain and Northern Ireland, the study found just under half (561 of 1194 individuals) of staff reported clinically significant anxiety and depression. 

As health systems struggle to cope with rising COVID-19 case numbers, many of them lacking the human and material resources they need, stakeholders ranging from hospital managers to civil society organizations are implementing measures designed to alleviate the pressure on health workers while increasing their capacity to cope.

“This is the worst situation we have ever faced.”

Flávia Machado

How best to achieve these different aims is a matter of debate. “At the moment we’re seeing a lot of different strategies to promote and protect the mental well-being of health workers, but good quality data and more research is needed to get a clear idea of how well those strategies actually work,” says Malik.

One of the biggest challenges faced is the lack of mental health workers in many countries, below two per 100 000 of population in low-income countries and a global median of nine per 100 000.

Reflecting this reality, guidance published by different agencies, including interim guidance published by WHO and the International Labour Organization in February 2021, emphasizes the importance of implementing changes in working conditions 

For some commentators this emphasis represents a helpful development. Among them is Dr Julian Eaton, a psychiatrist at the London School of Hygiene and Tropical Medicine. “We need to be careful about the idea that the only way that you support health-care workers is by providing counselling or psychological support,” he says, adding that one of the key findings of the *European Journal of Psychotraumatology* study was that lack of access to personal protective equipment increased the likelihood of mental distress. “It follows that making sure the health workers are protected is going to help their mental health,” he says.

Malik takes the same view. “Ensuring access to appropriate personal protective equipment or the knowledge on how to use it is not a psychosocial intervention per se but could have a huge impact on health workers’ mental health,” she says. 

The same argument is applied to reducing the workload of staff as much as possible. This decrease can be achieved by bringing in support, for example by bringing in workers from other sectors or specialties, reorganizing schedules to ensure regular staff rotation, or simply encouraging staff to take scheduled breaks. 

“Taking the trouble to ensure regular breaks for staff can make a huge difference,” says Eaton, pointing to a project implemented at the Miri General Hospital in Sarawak, Malaysia – a public tertiary hospital with 350 beds– as an example. 

“The hospital launched what they call their ‘24/7 Rest-’N-Go’ lounge at the beginning of the pandemic, a place where health-care workers involved in the COVID-19 response could rest in sanitized reclining chairs and also had access to free food, drinks and washrooms. It turned out to be extremely important for health-care workers to have a space where they could go and relax between and even during shifts,” Eaton says.

While such interventions may alleviate stress, what happens when a health worker needs to talk about something that may have happened to them?

For Malik, an important resource for health workers in managing the challenges they face is other health workers. “Peer-to-peer exchange can play a vital role,” she says, noting however, that several obstacles stand in the way of such exchanges, starting with health workers’ attitudes. 

“Individuals who have dedicated their careers to helping others tend not to be very good at seeking help for themselves,” she says. “They may also have a strong desire to appear stoic which causes them to hide any issues.” Workers are also reluctant to talk about issues that may be perceived by their colleagues or supervisors to affect their capacity to work.

Changing the culture that underpins such fears is clearly a long-term proposition. However, a start can be made by creating opportunities for discussion, for example by establishing procedures or mechanisms that allow for confidential discussion of experiences, challenges and dilemmas.

In the ongoing pandemic, peer-to-peer exchange is also proving itself valuable where specialist counselling is difficult to implement. In Pakistan for example, International Research and Development (IRD), a global health delivery and research organization, found that face-to-face counselling was unsuitable for the community health-care providers who are most of their employees.

“Fear of stigma is the number one obstacle to health-care workers speaking openly.”

Rawan Hamadeh

“At the beginning of the pandemic IRD’s mental health team implemented a system of face-to-face counselling,” explains Aneeta Pasha, Country Director of IRD Pakistan and Director of the IRD's mental health programme. “The problem was health-care workers took too much time to remove and put on again all the required personal protective equipment.”

IRD also set up a system whereby specialists called health workers to check on their mental well-being. According to Pasha, this too ran into trouble. “The calls were not always welcome as many of these professionals felt so exhausted at the end of their shifts that they prioritized sleep over talking about their mental health.” 

To get around these problems, IRD established an online community using a mobile-phone-based application. This allowed people to access discussions when they wanted to in a closed, non-judgemental environment. “The idea was to encourage our health-care providers to share their stories, and the feedback we have received so far is that members consider it an effective tool for reducing stress and learning coping strategies,” Pasha says.

The value of peer-to-peer exchange is also being demonstrated by the humanitarian aid organization Project HOPE, which is providing mental health support for health-care workers involved in the COVID-19 response in the Dominican Republic, Indonesia and the Philippines. 

According to Rawan Hamadeh, Project HOPE’s associate project coordinator for mental health programmes, the initiative uses both online and in-person sessions. The first session was held in September 2020 in Indonesia and 130 doctors, nurses and psychologists were brought together. “They openly discussed topics that are often silenced in the workplace, including exhaustion, depression, and compassion fatigue,” says Hamadeh, who says, “Fear of stigma is the number one obstacle to health-care workers speaking openly”. 

Her hope is that the initiatives will help reduce such obstacles. Machado believes that change may be coming anyway, under the relentless pressure of the pandemic. “It has become so much more common for people to have burnout and to feel exhausted and fragile that I have the impression that the stigma associated with talking about such things has been reduced,” she says. “Gradually, we are accepting this reality and discussing it more openly. This is a positive development.”

**Figure:**
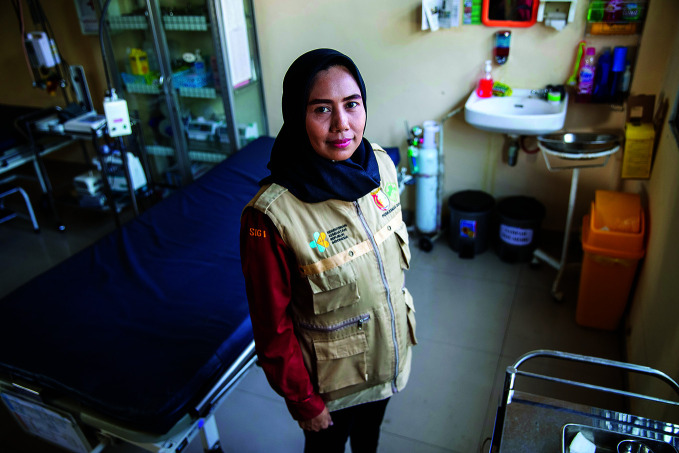
A participant in a peer-to-peer mental health resiliency initiative in Serang, Indonesia.

James Buck/Project HOPE

**Figure:**
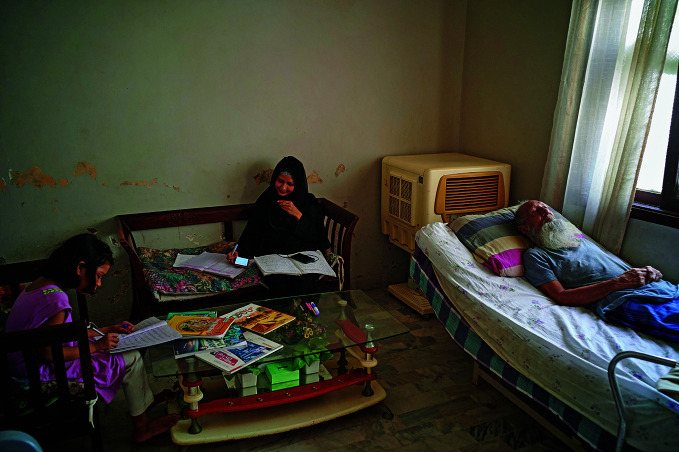
A mental health worker offers online counselling in Pakistan while caring for a sick parent.

Shehzad Noorani/IRD

